# Taking Cellular Heterogeneity Into Consideration When Modeling Astrocyte Involvement in Amyotrophic Lateral Sclerosis Using Human Induced Pluripotent Stem Cells

**DOI:** 10.3389/fncel.2021.707861

**Published:** 2021-09-17

**Authors:** Stefano Stifani

**Affiliations:** Department of Neurology and Neurosurgery, Montreal Neurological Institute-Hospital, McGill University, Montreal, QC, Canada

**Keywords:** astrocyte, induced pluripotent stem cells, heterogeneity, amyotrophic lateral sclerosis, disease modeling

## Abstract

Astrocytes are a large group of glial cells that perform a variety of physiological functions in the nervous system. They provide trophic, as well as structural, support to neuronal cells. Astrocytes are also involved in neuroinflammatory processes contributing to neuronal dysfunction and death. Growing evidence suggests important roles for astrocytes in non-cell autonomous mechanisms of motor neuron degeneration in amyotrophic lateral sclerosis (ALS). Understanding these mechanisms necessitates the combined use of animal and human cell-based experimental model systems, at least in part because human astrocytes display a number of unique features that cannot be recapitulated in animal models. Human induced pluripotent stem cell (hiPSC)-based approaches provide the opportunity to generate disease-relevant human astrocytes to investigate the roles of these cells in ALS. These approaches are facing the growing recognition that there are heterogenous populations of astrocytes in the nervous system which are not functionally equivalent. This review will discuss the importance of taking astrocyte heterogeneity into consideration when designing hiPSC-based strategies aimed at generating the most informative preparations to study the contribution of astrocytes to ALS pathophysiology.

## Introduction

Astrocytes are among the most abundant glial cell types in the mammalian nervous system (previously reviewed by Zuchero and Barres, 2015; Verkhratsky and Nedergaard, 2018). They contribute to a number of important physiological processes, such as trophic and structural support to neurons, synapse formation and maturation, plasticity, maintenance of the blood brain barrier, as well as neuroinflammatory mechanisms (previously reviewed by Wahis et al., 2020; Han et al., 2021; Tan et al., 2021). Together with microglia, astrocytes are the main sensors of nervous system injury and disease: they can undergo dramatic changes in their morphologies and functions in response to alterations in their environment, resulting in the acquisition of “reactive” phenotypes that can have both beneficial and detrimental effects on neighboring neuronal and glial cells (previously reviewed by Liddelow and Barres, 2017; Linnerbauer et al., 2020; Sofroniew, 2020). Reactive astrocytes mediate several biological responses, such as release of cytokines and chemokines involved in neuroinflammatory processes, intercellular communication with microglia and other neighboring cells, and modulation of immune responses (Liddelow and Barres, 2017; Linnerbauer et al., 2020; Sofroniew, 2020). Astrocyte activation is associated with numerous neurological conditions, including neurodevelopmental disorders and neurodegenerative diseases. Depending on the local microenvironment, reactive astrocytes are thought to contribute to either neuroprotective or neuroinflammatory responses (or both) (previously reviewed by Cresto et al., 2019; de Majo et al., 2020; Linnerbauer et al., 2020; Sofroniew, 2020).

## Involvement of Astrocytes in Amyotrophic Lateral Sclerosis

Amyotrophic lateral sclerosis (ALS) is a progressive and fatal neurodegenerative disease caused by death of motor neurons in the brain and spinal cord. Motor neuron degeneration in ALS results in progressive weakness and atrophy of multiple muscles, ultimately leading to loss of vital functions such as breathing. There is no effective treatment for ALS and most ALS patients die within 2 to 5 years following diagnosis (previously reviewed by Cook and Petrucelli, 2019; Mejzini et al., 2019; Kim et al., 2020; McAlary et al., 2020). The majority of ALS cases are sporadic, implying that the initial genetic causes and mechanisms triggering motor neuron loss are unknown. Approximately 10% of ALS cases are transmitted in families: these cases are referred to as familial ALS. The first genetic mutation to cause familial ALS, affecting the *SUPEROXIDE DYSMUTASE 1* (*SOD1*) gene, was described in 1993 (Rosen et al., 1993). Since then, multiple deleterious variants in many genes have been described that either drive motor neuron degeneration, increase susceptibility to the disease, or influence the rate of disease progression in ALS. Pathogenic mutations of the genes *TAR DNA BINDING PROTEIN* (*TARDBP)*, *FUSED IN SARCOMA/TRANLOCATED IN LIPOSARCOMA* (*FUS*), *OPTINEURIN* (*OPTN*), *TANK-BINDING KINASE 1* (*TBK1*), as well as intronic expansions in the gene *C9ORF72*, are among the most frequent familial ALS mutations (previously reviewed by Cook and Petrucelli, 2019; Mejzini et al., 2019; Kim et al., 2020; McAlary et al., 2020; Ustyantseva et al., 2020). Motor neurons display alterations of numerous cellular mechanisms in ALS, including RNA metabolism, protein homeostasis and aggregation, nucleocytoplasmic trafficking, endoplasmic reticulum stress, dynamics of ribonucleoprotein bodies, mitochondrial biology, and autophagy, to name a few (Mejzini et al., 2019; Kim et al., 2020; McAlary et al., 2020).

A number of previous studies suggest that motor neuron degeneration in ALS is not only caused by cell-autonomous cell death mechanisms but can also result from non-cell autonomous processes involving non-neuronal cells such as astrocytes and microglia (previously reviewed by Serio and Patani, 2018; Izrael et al., 2020; Vahsen et al., 2021; Van Harten et al., 2021). Evidence suggesting abnormal activation of astrocytes in ALS initially came from the analysis of *post-mortem* tissues, cerebrospinal fluids and blood samples from ALS patients (Schiffer et al., 1996; Poloni et al., 2000; Anneser et al., 2004; Baron et al., 2005), as well as the detection of marked neuroinflammation in the spinal cord and cranial motor nuclei in ALS transgenic mouse models (Schiffer et al., 1996; Ferraiuolo et al., 2007; Evans et al., 2014). Consistent with original *post mortem*/*in vivo* observations, several studies based on *in vitro* cultures of neural cells provided evidence that astrocytes harboring ALS-associated mutated proteins, such as mutated SOD1, are toxic to primary, as well as pluripotent stem cell-derived, motor neurons, but not to other neuronal populations (Di Giorgio et al., 2007, 2008; Nagai et al., 2007; Marchetto et al., 2008). These findings led to the suggestion that astrogliosis induced in response to initial insults to motor neurons gradually progresses from an initially neuroprotective role to neuroinflammatory effects that exacerbate neuronal degeneration (previously reviewed by Serio and Patani, 2018; Izrael et al., 2020; Vahsen et al., 2021). It is generally hypothesized that astrocytes exert deleterious effects on motor neuron in ALS as a consequence of either loss of supportive functions or gain of toxic functions (or a combination of both). Examples of loss of protective functions include disruption of the ability of astrocytes to regulate glutamate receptor 2 expression in motor neurons, rendering the latter more vulnerable to excitotoxicity (Van Damme et al., 2007). Examples of gain-of-function effects include production of reactive oxygen species causing motor neuron hyperexcitability and degeneration (Fritz et al., 2013; Rojas et al., 2015), inhibition of autophagy mechanisms in motor neurons (Tripathi et al., 2017), and extracellular vesicle-transmitted toxic microRNA species (Varcianna et al., 2019), to name only a few.

Although considerable advances in understanding the contribution of astrocytes to ALS pathophysiology has been made through the study of rodent models of ALS (see for instance Bruijn et al., 1997; Pardo et al., 2006; Cassina et al., 2008; Baker et al., 2015), there is growing evidence that these animal models cannot fully recapitulate human astrocyte phenotypes in ALS. Human and murine astrocytes differ at various levels, including morphology, function, and expression of genes enriched in disease-associated pathways (Chandrasekaran et al., 2016; Zhang et al., 2016; Kelley et al., 2018a; Hodge et al., 2019). These differences could be responsible, at least in part, for the sometime discordant results obtained using ALS rodent models, which have ranged from suggesting that astrocytes harboring ALS mutation have no direct roles in motor neuron degeneration, to proposing roles during disease progression, and even participation in triggering disease onset (e.g., Gong et al., 2000; Lino et al., 2002; Ferraiuolo et al., 2011; Wang et al., 2011). These observations have highlighted the importance of investigating the contribution of astrocytes to ALS pathophysiology using human cells.

The ability to study human astrocytes in health and disease has been limited by the difficulty in establishing primary cultures of human astrocytes, especially cells derived from adult patients affected by neurodegenerative diseases such as ALS. *Post mortem* human tissues, especially when subjected to high-throughput transcriptomic studies, can provide valuable insight into changes in gene expression occurring in the brain and spinal cord in neurodegenerative diseases (see for instance Al-Dalahmah et al., 2020; Lee H. et al., 2020). However, these studies are limited by the availability of tissues and are restricted to end-stages of the disease under study. Because of these considerations, increasing attention has focused on the application of human induced pluripotent stem cell (hiPSC)-based technologies toward the generation of human astrocytes. These approaches, combined with the power of gene-editing techniques to correct or introduce mutations, are providing previously unavailable experimental opportunities to address most of the above-mentioned shortcomings by generating human astrocyte preparations that can be used to study astrocyte biology, model astrocyte pathophysiological mechanisms, and support large-scale screens of new potential therapeutic compounds targeting astrocytes.

## Modeling the Roles of Human Astrocytes in Biology and Disease Using Human Induced Pluripotent Stem Cells

The past few years have seen a rapid growth in the number of reports describing hiPSC-based two- (2D) and three-dimensional (3D) protocols that can achieve robust and reliable generation of cells with molecular and functional properties resembling those of physiological astrocytes (e.g., Krencik and Zhang, 2011; Emdad et al., 2012; Shaltouki et al., 2013; Paşca et al., 2015; Chandrasekaran et al., 2016; Sloan et al., 2017; Tcw et al., 2017; Li et al., 2018; Zheng et al., 2018; Bradley et al., 2019; Leferink et al., 2019; Tchieu et al., 2019; Barbar et al., 2020; Ponroy Bally et al., 2020; Soubannier et al., 2020; Franklin et al., 2021, to list only a subset of published studies).

Astrocyte derivation from hiPSCs, whether in 2D or 3D approaches, commonly starts with the promotion of neural induction through inhibition of TGF-beta and bone morphogenetic protein (BPM) signaling pathways that act to prevent entry into the neural fate. Neural induction *in vitro* leads to the emergence of neural progenitor cells (NPCs) that no longer express typical iPSC markers and instead turn on the expression of genes that are expressed in NPCs *in vivo* during nervous system development. Common monolayer astrocyte derivation protocols achieve very efficient NPC generation in about 7–12 days (e.g., Tcw et al., 2017; Barbar et al., 2020; Soubannier et al., 2020). These uncommitted NPCs are then instructed to enter the astroglial cell lineage and undergo astrocyte differentiation and maturation by a variety of means, depending on whether differentiation is occurring in 2D or 3D preparations. Most commonly, NPCs are exposed to media containing strong astrocyte differentiation inducers, such as ciliary neurotrophic factor (CNTF), leukemia inhibitory factor (LIF), and/or BMP family proteins, leading to activation of JAK-STAT signaling pathways and other mechanisms promoting astrogenesis (reviewed in Chandrasekaran et al., 2016; Halpern et al., 2019; Lee K. M. et al., 2020; Franklin et al., 2021). The efficient *in vitro* generation of glial cells with molecular characteristics of astrocytes is then assessed by the combined expression of typical astrocytic markers, including *S100beta* (*S100B* using the human nomenclature), *AQUAPORIN-4* (*AQP4*), *CONNEXIN-43* (*GJA1*), and *ALDEHYDE DEHYDROGENASE 1 FAMILY MEMBER A1* (*ALDH1A1*), and the concomitant absence of expression of NPC, neuronal, and oligodendrocyte markers. Further validation involves bulk- and/or single cell (sc) RNA sequencing (RNAseq), combined with functional assays measuring spontaneous and induced calcium transients, glutamate uptake, and response to microglia-derived cytokines (e.g., Tcw et al., 2017; Leferink et al., 2019; Barbar et al., 2020; Soubannier et al., 2020). The specific features of different astrocyte differentiation protocols from hiPSCs will not be described hereafter because this topic has been extensively addressed in previous reviews (e.g., Chandrasekaran et al., 2016; Halpern et al., 2019; de Majo et al., 2020; Lee K. M. et al., 2020; Franklin et al., 2021). Instead, this review will focus on the importance of considering astrocyte heterogeneity when designing hiPSC-based differentiation strategies that will have the greatest potential to generate astrocytes relevant to the particular questions of ALS pathophysiology under study.

## Astrocyte Heterogeneity

During nervous system development, NPCs located at different AP and dorsal-ventral (DV) positions of the neural tube give rise to distinct populations of astrocytes that will perform specific biological functions in the mature nervous system (previously reviewed by Bayraktar et al., 2014; Tabata, 2015; Khakh and Deneen, 2019; de Majo et al., 2020). A number of astrocyte subtype-specific morphological and functional properties have long been recognized. Most notably, brain astrocytes with a fibrous morphology, characterized by long and relatively unbranched process, are typically found in the white matter. In contrast, astrocytes with a highly branched and bushy (protoplasmic) morphology are typically observed in the gray matter (Zhang and Barres, 2010; Oberheim et al., 2012; Bayraktar et al., 2014). These two main categories express different levels of the glial fibrillary acid protein (GFAP), namely high GFAP levels in fibrous astrocytes and lower levels in protoplasmic astrocytes. Further heterogeneity exists among the protoplasmic astrocyte group, which includes morphologically and molecularly distinct subtypes located in separate brain regions, where they selectively interact with distinct neuronal cells (Lanjakornsiripan et al., 2018; Bayraktar et al., 2020; Herrero-Navarro et al., 2021; Pembroke et al., 2021). Different types of astrocytes have also been described in the spinal cord, where their distribution along the DV axis is correlated with specialized interactions with particular neuronal neighbors, as a result of common patterning mechanisms during early stages of development (Hochstim et al., 2008; Tsai et al., 2012; Bayraktar et al., 2014; Molofsky et al., 2014). Thus, astrocyte identity is correlated with region-specific functions and cell type-specific interactions with neighboring neurons, as well as non-neuronal cells such as oligodendrocytes.

The biological heterogeneity of astrocytes in the healthy nervous system is, perhaps not surprisingly, correlated with the demonstration that not all astrocytes are equally susceptible to dysfunction in ALS, as well as other neurological conditions (Goursaud et al., 2009; Li et al., 2016; Bugiani et al., 2018; Köhler et al., 2021; Nguyen et al., 2021). Moreover, different types of astrocytes interact selectively with different motor neurons under both physiological and pathological conditions (Kelley et al., 2018a; Gomes et al., 2020; Mishra et al., 2020; Barbosa et al., 2021). More specifically, studies by Kelley and colleagues showed that loss of the inward-rectifying K^+^ channel Kir4.1 in astrocytes located in the ventral horn of the murine spinal cord selectively alters large fast alpha-motor neuron size and function, leading to reduced peak strength (Kelley et al., 2018b). Importantly, Kir4.1 is downregulated in astrocytes obtained from ALS patients with mutations in the *SOD1* gene, selectively implicating Kir4.1-expressing astrocyte dysfunction in ALS (Kelley et al., 2018b). Other studies have also suggested that astrocyte diversity is correlated with specific phenotypes in ALS, as exemplified by the observation that brain and spinal astrocytes from SOD1 (G93A) mice, a commonly used murine ALS model, display distinct inflammatory and neurotoxic phenotypes and impact differently on neuronal survival (Gomes et al., 2020; Barbosa et al., 2021). It is important to emphasize that regional differences in astrocytes were also associated with selective neurodegeneration in *in vitro* models of Parkinson’s Disease (PD). Isolated murine ventral tegmental area (VTA) and substantia nigra pars compacta astrocytes exhibited transcriptional differences consistent with regional diversity between these classes of astrocytes (Kostuk et al., 2019). More importantly, VTA astrocytes can play a role in the selective and subregional protection of VTA dopaminergic neurons from PD toxicity by releasing neuroprotective factors like GDF15 (Kostuk et al., 2019). Taken together, these observations underscore the importance of better understanding the specific roles of different astrocyte subtypes in both the healthy and diseased nervous systems.

## Taking Cellular Heterogeneity Into Consideration When Deriving Astrocytes From Human Induced Pluripotent Stem Cells for ALS Modeling

Modeling ALS using hiPSC-based approaches must include consideration of the *in vivo* heterogeneity of both neuronal and glial cells. Different subtypes of motor neurons are present along the anterior-posterior (AP) and medial-lateral (ML) axes of the central nervous system, and different motor neurons are not equally susceptible to degeneration in ALS (Nijssen et al., 2017; Ragagnin et al., 2019). Moreover, mechanisms of motor neuron death are not the same across different motor neuron subtypes during both development and disease (Tung et al., 2015; Mukaigasa et al., 2017; Morello et al., 2020). Thus, hiPSC-based motor neuron differentiation strategies should account for the regional and functional diversity of motor neurons. There is evidence that hiPSCs are competent to generate a repertoire of motor neuron subtypes with molecular signatures resembling those of motor neurons located in different AP and ML regions of the spinal cord (Thiry et al., 2020; Solomon et al., 2021). These observations raise hope that future ALS modeling studies utilizing motor neurons derived from hiPSCs can benefit from the ability to generate specific motor neuron subtypes with defined biological properties and specific mechanisms of degeneration in ALS.

As discussed above, the regional and functional diversity of astrocytes in both the brain and spinal cord underlies specialized interactions of specific astrocyte populations with particular neuronal neighbors (Hochstim et al., 2008; Tsai et al., 2012; Bayraktar et al., 2014; Molofsky et al., 2014; Kelley et al., 2018a; Mishra et al., 2020), underscoring the importance of accounting for astrocyte heterogeneity when studying the involvement of astrocytes in neurodegenerative diseases such as ALS. Up to date, hiPSC-derived astrocyte cultures used to model ALS pathophysiology have usually included a mix of multiple types of astrocytes (e.g., Hall et al., 2017; Madill et al., 2017; Qian et al., 2017; Tyzack et al., 2017; Kelley et al., 2018a; Birger et al., 2019; Varcianna et al., 2019; Rajpurohit et al., 2020; Smethurst et al., 2020; Zhao et al., 2020). This situation is not ideal to address the complexity of astrocyte dysfunction, and motor neuron-astrocyte communication, in ALS, especially when taking into consideration that multiple motor neuron subtypes are affected in ALS. There is growing evidence, however, suggesting that this limitation can be addressed. A number of studies have shown that hiPSCs are competent to give rise to different astrocyte subtypes with distinct molecular signatures and functional properties *in vitro* (Sloan et al., 2017; Li et al., 2018; Bradley et al., 2019; Kostuk et al., 2019; Tchieu et al., 2019). Moreover, methods have been described to instruct hiPSCs to generate astrocytes with region-specific properties, including GFAP-expressing astrocytes derived from anterior hindbrain-specific glial progenitor cells (Yun et al., 2019), as well as midbrain-patterned astrocytes (Booth et al., 2019). The ability of hiPSCs to give rise to glial cell diversity was also described in 3D cerebral organoids using scRNAseq approaches (Dang et al., 2021). It should be emphasized, however, that hiPSC-derived organoids have seen limited application to ALS modeling (reviewed in Ferraiuolo and Maragakis, 2021).

It is therefore reasonable to anticipate that it will become increasingly possible to include cellular heterogeneity in the considerations made when designing *in vitro* strategies to derive astrocytes from hiPSCs to model ALS, and possibly other neurodegenerative diseases with astrocyte pathologies. Improvements to *in vitro* differentiation strategies, combined with molecular characterization at the single-cell resolution, are expected to make it possible to generate those specific astrocyte subpopulations that are predicted to be most informative for the pathophysiological questions under study. A few relevant examples will be discussed in the following sections.

### White Matter or Gray Matter Astrocytes?

Astrocytes have the potential to contribute to ALS disease progression in several ways, including loss/reduction of trophic support, gain-of-function toxic effects on motor neurons in the gray matter and/or oligodendrocytes in the white matter, as well direct or indirect effects on motor neuron axon biology. Consistent with these possibilities, dysfunction of both gray matter and white matter astrocytes has been observed in ALS (Meadowcroft et al., 2015; Zou et al., 2019; Izrael et al., 2020; Killoy et al., 2020; Vahsen et al., 2021). Little progress has been made, however, in using hiPSC-derived astrocytes to study the specific contributions of either white matter or gray matter astrocytes to mechanisms of ALS pathophysiology, mainly because of the lack of derivation protocols that would give rise to cultures enriched for one cell type or the other. Leferink and coworkers have recently begun to address this shortcoming by describing a differentiation strategy that generates cultures enriched in white matter-like astrocytes from both human and mouse iPSCs (Leferink et al., 2019). Using differentiation media containing either CNTF (white matter) or fetal bovine serum (gray matter), they obtained distinct iPSC-derived astrocyte populations that differ in morphological characteristics and gene expression profiles *in vitro*. Importantly, only white matter-like astrocytes derived from hiPSCs showed vulnerability to mutations associated with leukodystrophy vanishing white matter, a selective white matter astrocyte disease (Bugiani et al., 2018). Moreover, only white matter-like astrocytes inhibited oligodendrocyte maturation *in vitro*, as is the case in vanishing white matter disease. Of note, human white matter-like astrocytes exhibited additional, human specific, phenotypes that were not observed in corresponding cells derived from mouse iPSCs (Leferink et al., 2019).

Other possible strategies to direct hiPSCs toward generating either gray matter or white matter astrocytes are suggested by lessons learned from *in vivo* studies. For instance, in the developing murine cerebral cortex, postnatal depletion of *OLIG2*+ neural progenitors, which mainly give rise to oligodendrocytes during gliogenesis, also results in a severe loss of white matter, but not gray matter, astrocytes (Cai et al., 2007; Tabata, 2015). Since *OLIG2* expression in NPCs is under the regulation of opposing ventralizing or dorsalizing cues, these observations suggest that a calibrated exposure of hiPSC-derived NPCs to DV patterning mechanisms might provide a way to enrich for gray matter-like astrocytes derived from *OLIG2*-negative (or *OLIG2*^*LOW*^) NPCs. The importance of carefully modulating DV patterning mechanisms in hiPSC-derived NPCs as a means of controlling astrocyte subtype differentiation is further suggested by the demonstration that separate progenitor domains competent to generate different classes of mouse astrocytes are present along the DV axis *in vivo* (Hochstim et al., 2008; Tsai et al., 2012). These concepts will be discussed more extensively below.

Although it is recognized that *in vitro* differentiation conditions may not fully recapitulate developmental processes *in vivo*, the described studies represent two examples of how combined knowledge of astrocyte development and biology and advances in hiPSC technology could be used toward the goal of enriching for gray matter or white matter astrocytes with distinct biological properties. In addition to the already-mentioned relevance to the study of vanishing white matter and other related diseases, the availability of cultures enriched in white matter-like astrocytes is expected to contribute to the study of specific ALS-relevant questions. For instance, even though reactive astrogliosis is widespread in the subcortical white matter of ALS patients (Kushner et al., 1991; Cardenas et al., 2017), the significance of this observation to myelinated axons and microglia-mediated neuroinflammation in the white matter remain to be fully characterized. Conversely, hiPSC-derived preparations enriched in gray matter-like astrocytes are predicted to be more informative when studying the involvement of astrocytes in the support, survival, and/or degeneration of selected motor neuron subtypes.

### Brain or Spinal Astrocytes?

Another important consideration that researchers should make when using hiPSC-derived astrocytes to model ALS, as well as other motor neuron diseases, is whether to choose derivation protocols starting from rostral (i.e., brain) or caudal (i.e., spinal) NPCs. This distinction is particularly important when studying mechanisms of selective astrocyte involvement in the degeneration of either upper (brain) or lower (brainstem and spinal cord) motor neurons in ALS. These neurons have different developmental origins, connectivities, functions and intrinsic mechanisms of degeneration (previously reviewed by Grad et al., 2017; Nijssen et al., 2017; Ragagnin et al., 2019). Thus, it seems likely that brain astrocytes would provide a better model than spinal astrocytes to study astrocyte involvement in upper motor neuron degeneration in ALS, and *vice versa* for lower motor neurons. It also important to note that motor neuron diseases such as primary lateral sclerosis and progressive muscular atrophy display predominant involvement of either upper motor neurons or lower motor neurons, respectively (Grad et al., 2017), underscoring the importance of carefully selecting which particular astrocyte subtypes to study in the context of these diseases.

During *in vivo* development, NPCs undergo AP and DV patterning through the combined activities of key signaling mechanisms, including retinoic acid (RA), fibroblast growth factor (FGF), SHH, BMP, and WNT pathways (Wichterle et al., 2002; Mallo and Alonso, 2013; Krumlauf, 2018; Leung and Shimeld, 2019; Sagner and Briscoe, 2019). Some of these mechanisms can be mimicked *in vitro* to generate rostral-like or caudal-like NPCs from hiPSCs, which in turn have the potential to give rise to brain-like or spinal cord-like astrocytes. The derivation of spinal astrocytes from hiPSCs is usually achieved using caudalized and ventralized NPCs (see for instance Roybon et al., 2013; Hall et al., 2017; Qian et al., 2017). NPCs derived from hiPSCs, as well as embryonic stem cells, appear to initially have a rostral identity, but a more caudal character can be imposed by exposure to controlled amounts of RA (Wichterle et al., 2002; Okada et al., 2004). The extent of caudalization can be assessed by monitoring specific *HOX* gene family members, such as *HOXC5*, *HOXC6*, *HOXC8*, whose expression is known to define spinal cord segments *in vivo* (Wichterle et al., 2002; Mallo and Alonso, 2013; Krumlauf, 2018). Ventralization is commonly achieved by activation of SHH signaling and can be validated by monitoring the combinatorial expression of typical spinal cord NPC markers, including *OLIG2*, *NKX6.1*, and *PAX6*, to name a few (Hochstim et al., 2008; Yu et al., 2013; Leung and Shimeld, 2019; Sagner and Briscoe, 2019). In contrast, brain astrocytes are usually generated from NPCs that are not exposed to RA, and often also in the absence of SHH agonists. Brain NPCs typically express rostral *HOX* genes, as well as brain-specific marker genes, such as *FOXG1* or *OTX1* (see for instance Shaltouki et al., 2013; Tcw et al., 2017; Ponroy Bally et al., 2020).

Although hiPSC-derived astrocytes generated from brain or spinal NPCs are generally considered functionally equivalent under *in vitro* conditions, there are a number of reasons to believe that they should not be equal. For instance, RA, which is used in caudalized preparations, facilitates LIF-induced astrocyte differentiation from NPCs, and contributes to the activation of the *GFAP* promoter through epigenetic changes (Asano et al., 2009). RA also promotes the formation of OLIG2-expressing oligodendrocyte precursor cells (OPCs) in the brain (Morrison et al., 2020). As mentioned above, these cells can also give rise to white matter astrocytes expressing high levels of *GFAP* (Cai et al., 2007; Tabata, 2015). Consistently, decreased numbers of OPCs result in decreased *GFAP* expression in the *corpus callosum* (Morrison et al., 2020). These observations suggest that NPCs treated with RA may be more prone to give rise to astrocytes expressing high levels of *GFAP*, a characteristic of white matter astrocytes. It should also be mentioned that increased levels of GFAP are a hallmark of reactive astrocytes (Zamanian et al., 2012; Liddelow et al., 2017). These factors should be considered when deciding whether or not to expose NPCs to RA (and what RA concentration to use).

The activation of SHH signaling as a ventralizing cue, used in conjunction with RA treatment, could also have important implications for astrocyte subtype generation. A gradient of SHH acts during spinal cord development to activate downstream mechanisms that establish a pattern of adjacent DV progenitor domains that give rise to separate neuronal and glial cells. In the same context, it is important to note that RA was shown to influence DV patterning during *in vitro* differentiation of mouse embryonic stem cells, namely higher concentrations of RA can induce more dorsal phenotypes than lower concentrations (Okada et al., 2004). Taken together, these findings recommend a careful consideration of the concentrations of SHH agonists and RA because of their effects on DV patterning. As will be described in more detail below, different subtypes of astrocyte are generated from separate DV progenitor domains in the spinal cord, and these different astrocytes preferentially interact with certain spinal neurons rather than others (Hochstim et al., 2008; Tsai et al., 2012). In conclusion, it is reasonable to assume that the properties of astrocytes generated *in vitro* from NPCs exposed to RA and SHH agonists will be different from those of astrocytes generated in the absence of either or both of them. Researchers should therefore carefully evaluate whether to utilize RA and/or SHH activation, calibrate the specific concentrations of these reagents, and assess AP (and DV) NPC marker expression, in order to design protocols predicted to give rise to the subtypes of rostral or caudal astrocytes that would be most appropriate for their specific studies.

Most, if not all, derivation protocols used thus far to generate astrocytes for ALS modeling have started from NPCs exposed to RA and SHH agonists (e.g., Hall et al., 2017; Qian et al., 2017; Kelley et al., 2018b; Birger et al., 2019; Varcianna et al., 2019; Zhao et al., 2020), suggesting that they likely gave rise to astrocytes with spinal features. This observation suggests that commonly used hiPSC-derived astrocyte preparations are suited for studying the involvement of astrocytes in non-cell autonomous mechanisms of lower motor neuron degeneration, but are not ideal for studies involving upper motor neurons. It is also reasonable to hypothesize that caudalized astrocyte preparations would be better suited when modeling progressive muscular atrophy (affecting predominantly lower motor neurons) than in the context of primary lateral sclerosis (upper motor neurons). Little information is available, however, about additional specific AP features (e.g., cervical, thoracic, or lumbar identity) of astrocyte preparations that have been used for ALS modeling. The opportunity to subject these preparations to single-cell biology studies is expected to address this shortcoming in future investigations. Taken together, these observations highlight the importance of using the most appropriate, and deeply phenotyped, astrocyte (and motor neuron) populations when modeling upper or lower motor neuron-astrocyte communication in ALS, or related motor neuron diseases, using hiPSCs.

### Dorsal or Ventral Astrocytes?

At least three subtypes of topologically and molecularly-defined white matter astrocytes were described in the ventral half of the developing mouse spinal cord (Hochstim et al., 2008). All of these cells express high levels of *GFAP*, but they occupy distinct locations along the DV and ML axes. These three white matter astrocyte subtypes derive from three separate ventral progenitor domains that arise in the spinal cord in response to graded SHH activity and can be distinguished through their combinatorial expression of the transcription factors NKX6.1 and PAX6 (Hochstim et al., 2008). A correlation between the topology of mature astrocytes and the DV position of their progenitors was also demonstrated for gray matter astrocytes. More specifically, spinal astrocytes localize to defined spatial domains in register with the DV position of their precursors in the ventricular zone (Tsai et al., 2012). Importantly, spinal cord astrocytes with different regional allocations become specialized for interactions with their own particular neuronal neighbors as a result of common patterning mechanisms *in vivo* (Tsai et al., 2012). Similar observations were made in the brain, where different subtypes of astrocytes present in different brain regions selectively modulate the functions of specific neuronal subtypes, which in turn can influence astrocyte functional diversity (Molofsky and Deneen, 2015; Bayraktar et al., 2020; Herrero-Navarro et al., 2021). Additional evidence of selective astrocyte-neuron subtype interactions along the DV axis is provided, as already discussed above, by the demonstration that ventral Kir4.1-dependent astrocyte-fast motor neuron interactions are required for maintenance of the cellular and biophysical properties of fast alpha-motor neurons (Kelley et al., 2018b).

Taken together, these observations underscore the importance of carefully optimizing ventralization conditions (e.g., concentration of SHH agonists and RA, and balance of canonical and non-canonical WNT signaling) that would favor ventral, versus dorsal, astrocyte differentiation. This would involve a thorough characterization of DV NPC marker expression, in order to select for the NPCs that would be predicted to give rise to astrocyte subtypes with the desired molecular properties, followed by validation of differentiated astrocytes using scRNAseq approaches. This has not been case in the field of ALS thus far, since common astrocyte differentiation protocols used for ALS modeling have usually relied on similar SHH and RA signaling conditions (e.g., Hall et al., 2017; Madill et al., 2017; Qian et al., 2017; Tyzack et al., 2017; Kelley et al., 2018b; Birger et al., 2019; Varcianna et al., 2019; Rajpurohit et al., 2020; Smethurst et al., 2020; Zhao et al., 2020).

The above considerations are particularly relevant when studying the involvement of astrocytes in the degeneration of spinal motor neurons, given the specific localization of these cells in the ventral spinal cord *in vivo*. In this regard, studies by Shijo and coworkers showed that BMP4 is upregulated in astrocytes in the ventral, but not dorsal, horn of the spinal cord of ALS SOD1 (H46R) transgenic rats. More importantly, inhibiting BMP4 resulted in decreased astrogliosis and attenuated motor dysfunction symptoms, highlighting a pathologically-relevant distinction between ventral and dorsal astrocytes in the context of ALS (Shijo et al., 2018).

In conclusion, when taken together with the examples discussed in the preceding subheadings, these observations provide several lines of evidence suggesting that modeling ALS using hiPSC-derived astrocytes and motor neurons will benefit from an increased attention to the choice of derivation protocols. Strategies optimized to generate specific subpopulations of astrocytes and neurons appropriate for the particular questions under study are expected to provide experimental model systems that will be more disease-relevant than generic preparations.

## Discussion

As discussed, a number of robust and reliable protocols are available to derive astrocytes from hiPSCs (previously reviewed by Chandrasekaran et al., 2016; Halpern et al., 2019; de Majo et al., 2020; Lee K. M. et al., 2020; Franklin et al., 2021). It is often the case that the choice of which protocol to use is made mainly on the basis of yield and time considerations. Although important, these factors are only a subset of the thoughts that should be given to the choice of the experimental conditions that will provide the most informative astrocyte preparations to address specific pathobiological questions in the context of ALS, especially when induced astrocytes are studied in co-cultures with motor neurons or other glial cells, as well as in the context of 3D preparations in which neuronal and glial cells differentiate together.

Careful design of astrocyte derivation protocols is particularly important in the case of ALS, because this disease affects a variety of brain and spinal cord motor neurons that are intrinsically different from one another and functionally interact with regionally-distinct subtypes of astrocytes. Thus, carefully choosing strategies yielding either brain or spinal astrocytes should be a necessary step when modeling astrocyte involvement in either upper or lower motor neuron degeneration in ALS. When studying spinal astrocytes, attention should also be paid to the AP identity (i.e., cervical, thoracic or lumbar) of the NPCs giving rise to astrocytes in order to optimize the experimental models established using hiPSCs.

The specific example of how to optimally model the roles of astrocytes in lower motor neuron degeneration in ALS using spinal hiPSC-derived astrocytes also highlights the importance of ensuring that astrocyte preparations have properties of ventral astrocytes. Dorsal spinal astrocytes would be less likely to functionally interact with motor neurons, even under *in vitro* conditions, and thus may not represent a disease-relevant model system. In this regard, the “ventral versus dorsal” consideration should also be important when deriving astrocyte to model other neurodegenerative diseases. For instance, Huntington’s Disease (HD), which is caused by polyglutamine-encoding CAG expansions in the first exon of the *HUNTINGTIN* (*HTT*) gene (MacDonald et al., 1993), is mainly characterized by loss of spiny motor neurons in the striatum, a ventral brain structure. This degeneration is hypothesized to occur because of the accumulation of misfolded mutated HTT protein in these neurons (Bates et al., 2015; Jimenez-Sanchez et al., 2017). Striatal astrocytes are also affected in HD, presenting with HTT aggregates similar to those observed in spiny motor neurons. This situation is correlated with marked astrogliosis in HD patients (Khakh et al., 2017). Moreover, studies using *in vivo* and *in vitro* models of the disease have shown that expression of mutated HTT in astrocytes contributes to neuronal excitotoxicity in HD (Shin et al., 2005; Bradford et al., 2010; Faideau et al., 2010). These findings implicate astrocytes in striatal neuron degeneration in HD. Based on these observations, it is reasonable to suggest that optimal modeling of the involvement of astrocytes in HD using hiPSCs from HD patients would benefit from the derivation of preparations enriched in ventral forebrain astrocytes.

Investigators should also consider aforehand whether they should aim at deriving induced cultures enriched in “gray versus white matter” astrocytes. In the context of ALS, this choice will depend on whether they plan, for instance, to investigate astrocyte-motor neuron or astrocyte-oligodendrocyte interactions, or mechanisms of neuroinflammation in the white matter or gray matter. Paying attention to this phenotypic trait when deriving astrocytes from hiPSCs should also be important when modeling other neurodegenerative diseases. For instance, PD is characterized by progressive loss of dopaminergic neurons in the substantia nigra pars compacta, which is thought to result in part from depositions of misfolded alpha-synuclein in neurons, resulting in neuronal toxicity (reviewed by Kalia and Lang, 2015; Shihabuddin et al., 2018). Importantly, alpha-synuclein accumulations were also observed in protoplasmic (i.e., gray matter) astrocytes, but not fibrous (i.e., white matter) astrocytes, in PD patients (Braak et al., 2007; Song et al., 2009). Importantly, protoplasmic astrocytes that accumulated alpha-synuclein in PD cases did not appear to be activated, whereas deposition of alpha-synuclein in protoplasmic astrocytes coincided with hallmarks of astrogliosis in brains from patients affected by another parkinsonian syndrome, progressive supranuclear palsy (Song et al., 2009). These observations further suggest that different types of astrocytes respond differently to common pathogenic mechanisms, providing further evidence of the importance of generating the most relevant subpopulations when studying specific astrocyte contributions to neurodegenerative diseases.

In conclusion, designing the most appropriate hiPSC-based astrocyte derivation strategies is expected to become increasingly important when modeling complex mechanisms of neurodegeneration. This goal will be facilitated by the growing understanding of the mechanisms governing the generation of astrocyte diversity *in vivo*, and of the specific functions performed by different astrocyte subtypes. The combination of increased knowledge of biological mechanisms, advances in hiPSC technologies, availability of high-throughput transcriptomic and proteomic approaches, and increasingly refined monolayer or 3D preparations is expected to offer unprecedented opportunities to clarify the specific roles of astrocytes in the pathophysiology of ALS, as well as other neurodegenerative diseases.

## Author Contributions

The author confirms being the sole contributor of this work and has approved it for publication.

## Conflict of Interest

The author declares that the research was conducted in the absence of any commercial or financial relationships that could be construed as a potential conflict of interest.

## Publisher’s Note

All claims expressed in this article are solely those of the authors and do not necessarily represent those of their affiliated organizations, or those of the publisher, the editors and the reviewers. Any product that may be evaluated in this article, or claim that may be made by its manufacturer, is not guaranteed or endorsed by the publisher.
